# The predictive value of platelet parameters for the response to initial ^131^I therapy in patients with differentiated thyroid cancer

**DOI:** 10.3389/fendo.2026.1850386

**Published:** 2026-06-19

**Authors:** Xian Wang, Suyun Yang

**Affiliations:** 1 Academy of Medical Sciences, Shanxi Medical University, Taiyuan, Shanxi, China; 2 School of Nursing, Shanxi Medical University, Taiyuan, Shanxi, China; 3 Department of Nuclear Medicine, First Hospital of Shanxi Medical University, Taiyuan, Shanxi, China; 4 Collaborative Innovation Center for Molecular Imaging of Precision Medicine, Taiyuan, Shanxi, China

**Keywords:** ^131^I, differentiated thyroid cancer, platelets, predictive value, treatment response

## Abstract

**Objective:**

This study aims to systematically evaluate the predictive value of platelet parameters (PLT, PDW, MPV, PCT) before initial ^131^I therapy in patients with differentiated thyroid cancer.

**Methods:**

This retrospective study included 365 patients with differentiated thyroid cancer (DTC) who underwent initial ^131^I therapy at the Department of Nuclear Medicine in a tertiary hospital. Treatment response was assessed according to the 2025 American Thyroid Association Guidelines for the Management of Adult Differentiated Thyroid Cancer, and was categorized as Excellent Response (ER) or Non-Excellent Response (non-ER). Univariate and multivariate analyses were performed to examine the association between PLT, PDW, MPV, and PCT with non-ER, adjusting for confounding factors. Results are presented as odds ratios (OR) and 95% confidence intervals (CI). The stability of the results was verified through interaction tests and subgroup analyses. Receiver operating characteristic (ROC) curves were plotted, and the area under the curve (AUC) was calculated to assess the predictive value of platelet indices for treatment response.

**Results:**

After adjusting for confounding factors, MPV was independently and negatively associated with non-ER (OR = 0.40, 95% CI 0.28–0.58). Interaction analysis suggested that age and ps-Tg influence the association between MPV and non-ER (*P* = 0.042 and *P* = 0.031). The ROC curve showed that MPV had a moderate predictive value for non-ER (AUC = 0.664). Based on the maximum Youden index, the optimal cutoff value was determined to be 9.95 fL, with a sensitivity of 0.595 and a specificity of 0.657.

**Conclusion:**

MPV demonstrated moderate predictive value in forecasting the response of DTC patients to initial ^131^I therapy (AUC = 0.664). In clinical practice, MPV may be of adjunctive value in helping to identify patients with poor treatment response at an early stage. However, it is insufficient to be used as the sole basis for clinical decision-making.

## Introduction

Thyroid cancer (TC), the most common malignant tumor of the head and neck, originates from the follicular or parafollicular epithelial cells of the thyroid gland (1). According to global cancer statistics for 2022, thyroid cancer ranked seventh in incidence among all cancers, and its incidence continues to increase worldwide (2, 3). Differentiated thyroid cancer (DTC) is the primary subtype, accounting for more than 90% of thyroid cancers (1). ^131^I therapy is the primary treatment for DTC. Although the overall prognosis for most DTC patients is favorable, approximately 20% of patients may still experience recurrence and distant metastasis. Among these patients, about two-thirds will eventually progress to radioactive iodine-refractory differentiated thyroid cancer (RAIR-DTC), a condition associated with a poor prognosis. Clinical data indicate that the median survival is less than 5 years, and the 10-year survival rate is below 10% (4, 5). On the other hand, studies suggest that at least one in five low-risk DTC patients may have received unnecessary or excessive radioactive iodine therapy (6). Overtreatment may increase the risk of radioactive iodine-related adverse reactions, such as impaired taste and smell, sialadenitis, and an increased long-term risk of secondary malignancies (7). Therefore, identifying biomarkers that can predict treatment response to ^131^I therapy in DTC patients is important for the early identification of high-risk individuals and the optimization of treatment strategies.

The 2025 American Thyroid Association (ATA) Guidelines emphasize that the response to initial ^131^I therapy is a key factors influencing clinical management decisions (8). The guidelines highlight the importance of early assessment of treatment response early in the initial therapy phase to determine the frequency and type of follow-up examinations. Therefore, it is essential to identify and predict patients who may have poor treatment response as early as possible to enable personalized care and treatment. This approach aims to reduce overtreatment and unnecessary use of medical resources while ensuring efficacy and safety.

Several studies have identified various potential predictors of treatment response to ^131^I therapy in patients with DTC, including tumor size (9), pre-stimulated thyroglobulin (ps-Tg) (10, 11), extrathyroidal extension, extracapsular invasion of lymph nodes (12), pre-treatment TSH level (13), ATA risk of recurrence (14), and TNM stage (15). Previous studies have shown that ps-Tg has good clinical predictive value. But there is no consensus on its clinical cutoff value. The 2025 ATA Guidelines note that most laboratories currently use immunoassays to measure Tg. This method is susceptible to interference from thyroglobulin antibodies (TgAb), which typically results in falsely low serum Tg levels. At present, there is no reliable method to completely eliminate the influence of TgAb (8). These issues have restricted the clinical application of ps-Tg in assessing the efficacy of ^131^I therapy in patients with DTC to some extent.

Therefore, additional predictive markers are needed to complement existing clinicopathological and biochemical indicators and to further improve the assessment of treatment response to initial ^131^I therapy in patients with DTC. During thyroid cancer progression, platelets not only participate in coagulation and thrombosis but may also contribute to tumor microenvironment remodeling, angiogenesis, immune evasion, and tumor progression by releasing pro-inflammatory factors, chemokines, and various growth factors (16). The complex interactions between platelets and tumor cells constitute a key mechanism underlying hematogenous metastasis. Tumor cells induce platelet aggregation, and platelets assist tumor cells in infiltrating blood vessels to facilitate hematogenous metastasis. Circulating tumor cells can further activate platelets. Platelets may protect tumor cells from immune attack and promote tumor cell proliferation, tumor microenvironment remodeling, and metastatic lesion formation (17, 18). In recent years, platelet-related parameters, including platelet count (PLT), platelet distribution width (PDW), mean platelet volume (MPV), and plateletcrit (PCT), have received increasing attention in thyroid cancer research. These parameters may reflect tumor-associated inflammation and aggressive biological behavior in thyroid cancer and may have potential value in predicting treatment response to initial ^131^I therapy.

Accordingly, it is of great significance to explore new predictive markers in order to overcome the limitations of existing ones and more accurately evaluate the treatment response to initial ^131^I therapy in patients with DTC.

## Methods

### Study population

**Figure 1:**
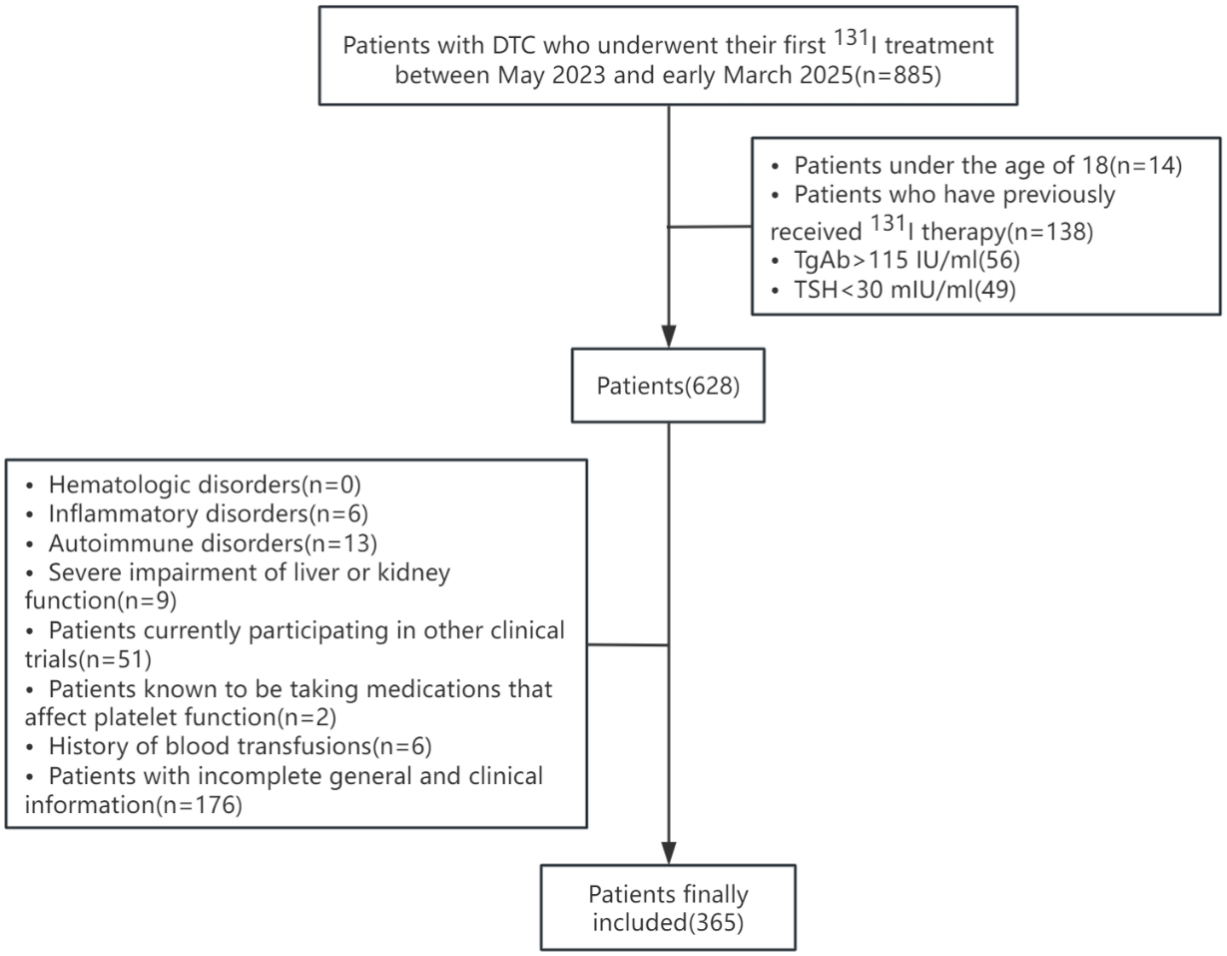
Flowchart of patient inclusions and exclusions in the study.

This retrospective study included 365 patients with DTC who underwent initial ^131^I therapy at the Department of Nuclear Medicine, First Hospital of Shanxi Medical University, between May 2023 and March 2025. The inclusion criteria were as follows: (1) age ≥ 18 years; (2) pathologically confirmed DTC and receipt of initial ^^131^I therapy after thyroidectomy; (3) TgAb < 115 IU/mL and TSH > 30 mIU/mL before the initial ^131^I treatment; (4) Patients with complete demographic and clinical data. The exclusion criteria were as follows: (1) Patients with hematological diseases; (2) Inflammatory diseases; (3) Autoimmune diseases; (4) Severe hepatic or renal impairment; (5) Patients currently participating in other clinical trials; (6) Patients known to be using medications that affect platelets; (7) Patients with a history of blood transfusion; (8) Patients with incomplete general or clinical information. The participant flow chart is shown in **Figure 1**.

### Outcome ascertainment

In this study, the assessment of treatment response to ^131^I therapy in patients with DTC was assessed according to the 2025 ATA Guidelines for the Management of Adult Differentiated Thyroid Cancer (8). Assessments were based on serological and imaging findings obtained 6–12 months after ^131^I therapy. Patients were categorized into two groups: excellent response (ER) and non-excellent response (non-ER). The non-ER group included Biochemical Incomplete Response (BIR), Structural Incomplete Response (SIR), and Indeterminate Response (IDR).

### Data collection

The following demographic characteristics and clinical data were collected on the first day of hospitalization before ^131^I therapy: age, sex, BMI, ^131^I dose (mCi), interval between surgery and the initial ^131^I treatment, tumor size, cancer position, extrathyroidal extension, multifocal tumor, lymph node metastasis, number of metastatic lymph nodes, tumor T stage, AJCC classification, ATA recurrence risk stratification, diabetes, hypertension, cardiovascular disease (CVD), TPOAb (IU/mL), TgAb (IU/mL), ps-Tg (ng/mL), PLT (*10⁹/L), PDW (fL), MPV (fL), PCT (%).

### Statistical analysis

All statistical analyses were performed using SPSS 27.0 and R software (version 4.3.2). Missing data were handled using the complete-case analysis method, and patients with missing values for key variables were excluded from the final analysis. Categorical variables were presented as frequencies (n) and percentages (%), and differences between groups were compared using the chi-squared test. Continuous variables that followed a normal distribution were described as mean ± standard deviation (SD), and comparisons between groups were performed using the independent-samples t-test. Continuous variables that did not follow a normal distribution were described using the median and interquartile range [M (P25, P75)], and differences between groups were compared using the Mann-Whitney U test. Univariate analysis was conducted to identify factors associated with the response to ^131^I therapy. Before constructing the multivariable regression models, multicollinearity among candidate variables was assessed using tolerance and variance inflation factors (VIFs). Variables showing severe multicollinearity were not included simultaneously in the same adjusted model. Multivariable binary logistic regression analysis was used to assess the associations of PLT, PDW, MPV, and PCT with treatment response, after adjustment for potential confounders. Results are presented as odds ratios (OR) and 95% confidence intervals (CIs). To assess the robustness of the results, interaction tests and subgroup analyses were performed, according to sex, age, BMI, ^131^I dose, number of lymph node metastases, tumor size, and ps-Tg. Receiver operating characteristic (ROC) curves were constructed to evaluate the predictive performance of the indicators, and the area under the curve (AUC) was calculated. The optimal cutoff value was determined using the maximum Youden index, and sensitivity, specificity, and their 95% CIs were further calculated. Decision curve analysis (DCA) was performed to assess the clinical utility of the predictive indicator. *P*-value of <0.05 was considered statistically significant.

## Results

### Characteristics of participants

**Figure 2:**
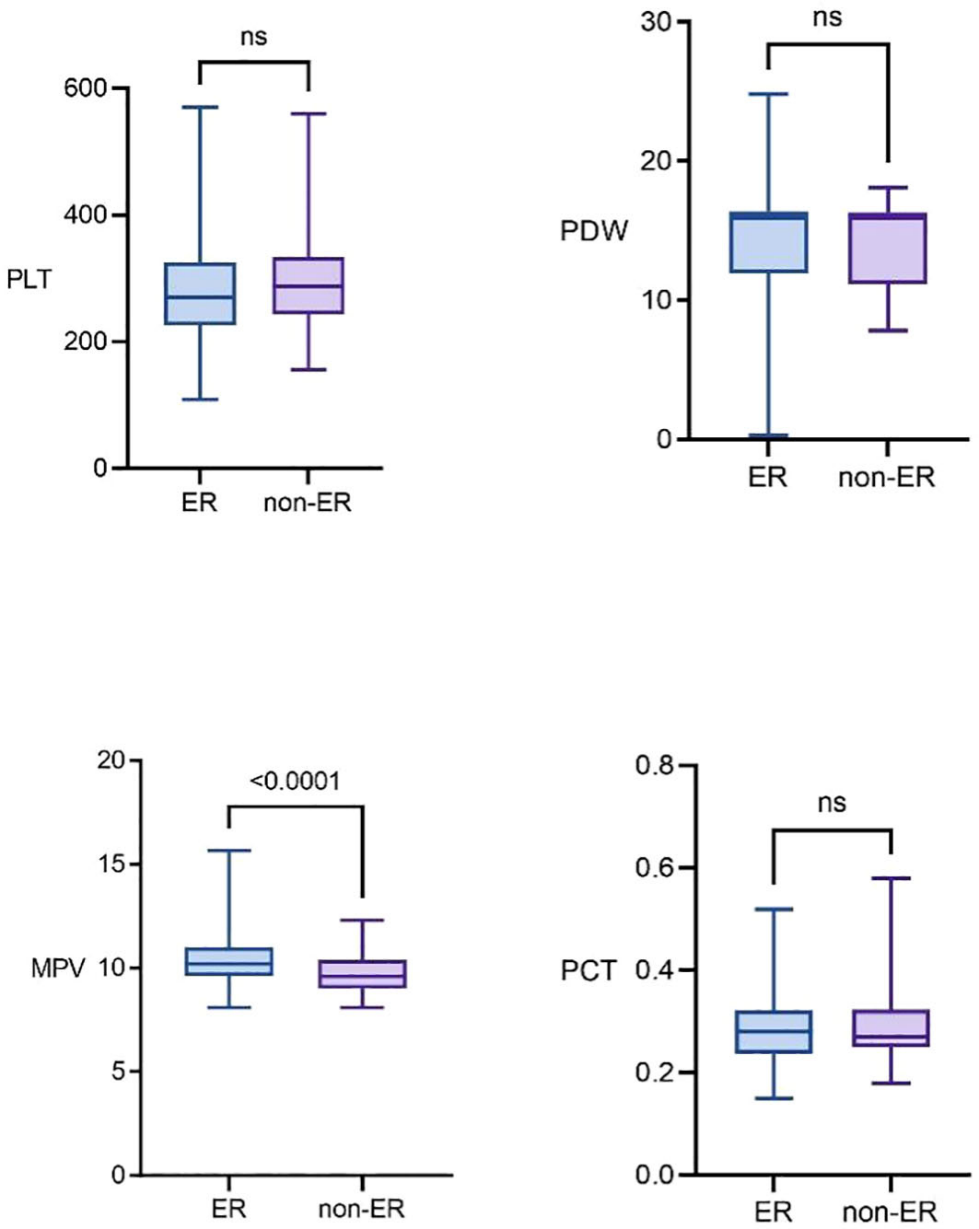
Boxplots of PLT, PDW, MPV, and PCT in the ER and non-ER groups. (ns: no statistically significant difference. The center line within the box indicates the median, the box represents the interquartile range, and the whiskers indicate the minimum and maximum values.).

This study included a total of 365 patients with DTC who were undergoing initial ^131^I therapy, including 257 patients in the ER group (70.41%) and 108 patients in the non-ER group (29.59%). Significant differences were observed between the two groups in terms of ^131^I treatment dose, tumor size, number of lymph node metastases, T stage, ATA recurrence risk stratification, hypertension, TgAb, ps-Tg, and MPV (*P* < 0.05). The results are shown in **Table 1**. The distribution of platelet parameters between the ER group and the non-ER group is shown in **Figure 2**. The distribution of ER and non-ER subcategories in the study cohort is shown in **Supplementary Table 1**.

**Table 1:** Comparison of baseline data between the ER and non-ER groups.

Variables	ER(n = 257)	Non-ER(n = 108)	P
Age	43.89 ± 10.68	42.94 ± 13.27	0.259
Sex			0.146
Male	175(47.95%)	65(17.81%)	
Female	82(22.47%)	43(11.78%)	
BMI	25.50 ± 3.88	25.45 ± 4.23	0.864
^131^I dose (mCi)	122.26 ± 19.27	144.21 ± 26.05	<0.001
Time interval (month)	2.35 ± 2.01	2.51 ± 2.09	0.866
Tumor size (cm)	1.10(0.70, 1.60)	1.50(1.00, 2.50)	<0.001
Cancer position			0.628
Unilateral	138(37.81%)	55(15.07%)	
Bilateral	119(32.60%)	53(14.52%)	
Extrathyroidal extension			0.099
No	210(57.53%)	80(21.92%)	
Yes	47(12.88%)	28(7.67%)	
Multifocal cancer			0.883
No	171(46.85%)	71(19.45%)	
Yes	86(23.56%)	37(10.14%)	
Lymph node metastasis			0.234
N0	19(5.21%)	3(0.82%)	
N1a	72(19.73%)	33(9.04%)	
N1b	166(45.48%)	72(19.73%)	
Number of lymph node metastasis	6.00(3.00, 11.00)	10(5.25, 16.75)	<0.001
T stage			<0.001
T1	193(52.88%)	56(15.34%)	
T2	22(6.03%)	23(6.30%)	
T3	30(8.22%)	16(4.38%)	
T4	12(3.29%)	13(3.56%)	
AJCC			0.231
I	225(61.64%)	91(24.93%)	
II	29(7.95%)	12(3.29%)	
III	2(0.55%)	3(0.82%)	
IV	1(0.27%)	2(0.55%)	
ATA risk of recurrence			<0.001
Low risk	6(1.64%)	0(0.00%)	
Intermediate risk	234(64.11%)	86(23.56%)	
High risk	17(4.66%)	22(6.03%)	
Diabetes			0.678
No	241(66.03%)	100(27.40%)	
Yes	16(4.38%)	8(2.19%)	
Hypertension			<0.001
No	227(62.19%)	80(21.92%)	
Yes	30(8.22%)	28(7.67%)	
CVD			0.953
No	252(69.04%)	106(29.04%)	
Yes	5(1.37%)	2(0.55%)	
TPOAb (IU/ml)	14.03(9.31, 28.00)	12.62(9.26, 18.04)	0.128
TgAb (IU/ml)	15.99(13.90, 24.23)	14.44(12.09, 16.50)	<0.001
ps-Tg(ng/mL)	0.96(0.20, 2.54)	15.68(5.13, 32.51)	<0.001
PLT(*109/L)	284.10 ± 80.72	294.25 ± 67.23	0.073
PDW (fL)	14.39 ± 2.79	14.25 ± 2.81	0.559
MPV (fL)	10.33 ± 1.34	9.63 ± 1.33	<0.001
PCT (%)	0.29 ± 0.07	0.28 ± 0.06	0.710

### Association of platelet parameters with treatment response

Univariate logistic regression analysis was performed to identify factors associated with the response to initial ^131^I therapy in patients with DTC. The results showed that ^131^I dose, tumor size, number of lymph node metastases, T stage, hypertension, TgAb, ps-Tg, PLT (Q3), MPV, and PCT (Q2) were significantly associated with treatment response (*P* < 0.05). The results are shown in **Table 2**.

**Table 2:** Univariate analysis.

Variables	N(%)/Mean ± SD	OR(95%CI)	P value
Age	43.61 ± 11.50	0.99(0.97, 1.01)	0.474
Sex			
Male	125(34.25%)	1.0	
Female	240(65.75%)	1.41(0.89, 2.25)	0.147
BMI	25.48 ± 3.98	1.00(0.94, 1.06)	0.917
^131^I dose (mCi)	128.75 ± 23.69	1.05(1.04, 1.06)	<0.001
Time interval (month)	2.40 ± 2.04	1.04(0.94, 1.15)	0.482
Tumor size (cm)	1.47 ± 1.00	1.69(1.34, 2.12)	<0.001
Cancer position			
Unilateral	193(52.88%)	1.0	
Bilateral	172(47.12%)	0.90(0.57, 1.40)	0.628
Extrathyroidal extension			
No	290(79.45%)	1.0	
Yes	75(20.55%)	1.56(0.92, 2.67)	0.101
Multifocal cancer			
No	242(66.30%)	1.0	
Yes	123(33.70%)	1.04(0.65, 1.67)	0.883
Lymph node metastasis			
N0	22(6.03%)	1.0	
N1a	105(28.77%)	2.90(0.80, 10.50)	0.104
N1b	138(38.81%)	2.75(0.79, 9.58)	0.113
Number of lymph node metastasis	9.04 ± 7.40	1.07(1.04, 1.10)	<0.001
T stage			
T1	249(68.22%)	1.0	
T2	45(12.33%)	3.60(1.87, 6.94)	<0.001
T3	46(12.60%)	1.84(0.94, 3.61)	0.077
T4	25(6.85%)	3.73(1.61, 8.64)	0.002
AJCC			
I	316(86.58%)	1.0	
II	41(11.23%)	1.02(0.50, 2.09)	0.950
III	5(1.37%)	3.71(0.61, 22.57)	0.155
IV	3(0.82%)	4.95(0.44, 55.21)	0.194
ATA risk of recurrence			
Low risk	6(1.60%)	1.0	
Intermediate risk	320(87.67%)	Inf. (0.00, Inf)	
High risk	39(10.68%)	Inf. (0.00, Inf)	
Diabetes			
No	341(93.42%)	1.0	
Yes	24(6.58%)	1.21(0.50, 2.91)	0.678
Hypertension			
No	307(84.11%)	1.0	
Yes	58(15.89%)	2.65(1.49, 4.71)	<0.001
CVD			
No	358(98.08%)	1.0	
Yes	7(1.92%)	0.95(0.18, 4.98)	0.953
TPOAb (IU/ml)	30.14 ± 92.74	0.99(0.97, 1.00)	0.190
TgAb (IU/ml)	22.88 ± 21.42	0.97(0.96, 0.99)	0.003
ps-Tg(ng/mL)	14.36 ± 54.60	1.07(1.04, 1.09)	<0.001
PLT(*109/L)	287.10 ± 77.03	1.00(1.00, 1.01)	0.251
Q1	93(25.48%)	1.0	
Q2	91(24.93%)	1.31(0.66, 2.58)	0.440
Q3	89(24.38%)	2.35(1.23, 4.49)	0.010
Q4	92(25.21%)	1.68(0.86, 3.26)	0.129
PDW (fL)	14.35 ± 2.80	0.98(0.91, 1.06)	0.657
Q1	101(27.67%)	1.0	
Q2	73(20.00%)	0.66(0.35, 1.23)	0.189
Q3	93(25.48%)	1.02(0.56, 1.85)	0.960
Q4	98(26.85%)	0.70(0.36, 1.39)	0.309
MPV (fL)	10.13 ± 1.24	0.56(0.44, 0.71)	<0.001
Q1	101(27.67%)	1.0	
Q2	86(23.56%)	0.53(0.29, 0.96)	0.036
Q3	95(26.03%)	0.33(0.18, 0.61)	<0.001
Q4	83(22.74%)	0.21(0.11, 0.43)	<0.001
PCT (%)	0.29 ± 0.07	0.29(0.01, 8.50)	0.475
Q1	92(25.21%)	1.0	
Q2	94(25.75%)	1.89(1.01, 3.55)	0.047
Q3	90(24.66%)	0.92(0.47, 1.79)	0.798
Q4	89(24.38%)	1.25(0.66, 2.39)	0.499

Multivariable logistic regression analysis was performed to assess the independent associations of PLT, PDW, MPV, and PCT with treatment response to initial ^131^I therapy in patients with DTC. These platelet indices were analyzed both as continuous variables and as quartile-based categorical variables. Before constructing the multivariate models, we assessed multicollinearity using tolerance and VIFs (**Supplementary Table 2**). The results showed that most variables did not exhibit severe multicollinearity. However, the VIF values for PLT and PCT were 24.247 and 20.357, respectively, indicating severe multicollinearity between these two variables. Therefore, in the final adjusted model, PLT and PCT were not included simultaneously in the same adjusted model. Variables included in the adjusted model were selected based on their clinical relevance, prior evidence, and potential roles as confounders, rather than solely on statistical significance in univariate analyses. The results showed that in the unadjusted model, patients in the PLT Q3 group had 2.35-fold higher odds of non-ER than those in the Q1 group. For each 1-fL increase in MPV as a continuous variable, the odds of non-ER decreased by 44%. When analyzed as a quartile, the odds of non-ER in the MPV Q2 group were 47% lower than in the Q1 group, 67% lower in the Q3 group, and 79% lower in the Q4 group. The odds of non-ER were 1.89-foldhigher in the PCT Q2 group than in the Q1 group. After full adjustment for potential confounders in Model 3, only MPV, both as a continuous and a quartile variable, remained significantly associated with treatment response. For each 1-fL increase in MPV, the odds of non-ER occurrence decreased by 60%. Compared with the MPV Q1 group, the odds of non-ER were 68%, 89%, and 96% lower in the MPV Q2, Q3, and Q4 groups, respectively. The results are shown in **Table 3**.

**Table 3:** Association of PLT, PDW, MPV, PCT and ^131^I treatment response.

Exposure	Unadjusted Model OR(95%CI)P	Model 1 OR(95%CI)P	Model 2 OR(95%CI)P	Model 3 OR(95%CI)P
PLT	1.00(1.00, 1.01)0.251	1.00(1.00, 1.01)0.135	1.00(1.00, 1.01)0.159	1.00(1.00.1.01)0.382
Q1	1.0	1.0	1.0	1.0
Q2	1.31(0.66, 2.58)0.440	1.32(0.66, 2.62)0.429	1.86(0.82, 4.19)0.137	2.24(0.91, 5.51)0.080
Q3	2.35(1.23, 4.49)0.010	2.57(1.32, 5.00)0.005	2.49(1.10, 5.65)0.029	2.24(0.91, 5.50)0.079
Q4	1.68(0.86, 3.26)0.129	1.89(0.94, 3.79)0.073	2.34(1.02, 5.37)0.045	2.07(0.83, 5.17)0.117
PDW	0.98(0.91, 1.06)0.657	0.98(0.91, 1.07)0.671	0.95(0.86, 1.04)0.248	0.94(0.84, 1.04)0.223
Q1	1.0	1.0	1.0	1.0
Q2	0.66(0.35, 1.23)0.189	0.66(0.35, 1.24)0.196	0.60(0.29, 1.24)0.164	0.48(0.21, 1.08)0.076
Q3	1.02(0.56, 1.85)0.960	1.03(0.56, 1.89)0.918	0.77(0.38, 1.58)0.483	0.67(0.29, 1.59)0.369
Q4	0.70(0.36, 1.39)0.309	0.68(0.34, 1.35)0.268	0.47(0.21, 1.09)0.079	0.41(0.16, 1.05)0.062
MPV	0.56(0.44, 0.71)<0.001	0.54(0.42, 0.69)<0.001	0.49(0.36, 0.66)<0.001	0.40(0.28, 0.58)<0.001
Q1	1.0	1.0	1.0	1.0
Q2	0.53(0.29, 0.96)0.036	0.52(0.28, 0.95)0.032	0.45(0.22, 0.91)0.026	0.32(0.12, 0.88)0.028
Q3	0.33(0.18, 0.61)<0.001	0.30(0.16, 0.56)<0.001	0.21(0.10, 0.45)<0.001	0.11(0.03, 0.41)<0.001
Q4	0.21(0.11, 0.43)<0.001	0.20(0.10, 0.41)<0.001	0.14(0.06, 0.35)<0.001	0.04(0.01, 0.26)<0.001
PCT	0.29(0.01, 8.50)0.475	0.41(0.01, 14.92)0.623	0.33(0.01, 22.97)0.611	0.04(0.00, 4, 58)0.180
Q1	1.0	1.0	1.0	1.0
Q2	1.89(1.01, 3.55)0.047	1.98(1.03, 3.78)0.040	2.02(0.95, 4.30)0.067	2.27(0.96, 5.37)0.062
Q3	0.92(0.47, 1.79)0.789	0.96(0.48, 1.89)0.897	0.85(0.38, 1.94)0.705	0.90(0.37, 2.20)0.814
Q4	1.25(0.66, 2.39)0.499	1.40(0.70, 2.80)0.339	1.27(0.56, 2.88)0.565	0.87(0.35, 2.18)0.763

Model 1 was adjusted for sex, age, and BMI. Model 2 was adjusted for sex, age, BMI, ^131^Idose, tumor size, number of lymph node metastases, and ps-Tg. Model 3 was adjusted for sex, age, BMI, ^131^I dose, tumor size, number of lymph node metastases, ps-Tg, time interval between surgery and initial ^131^I therapy, tumor position, extrathyroid extension, multifocality, lymph node metastasis stage, T stage, AJCC, ATA recurrence risk stratification, diabetes, hypertension, cardiovascular disease, TPOAb, and TgAb.

**Figure 3:**
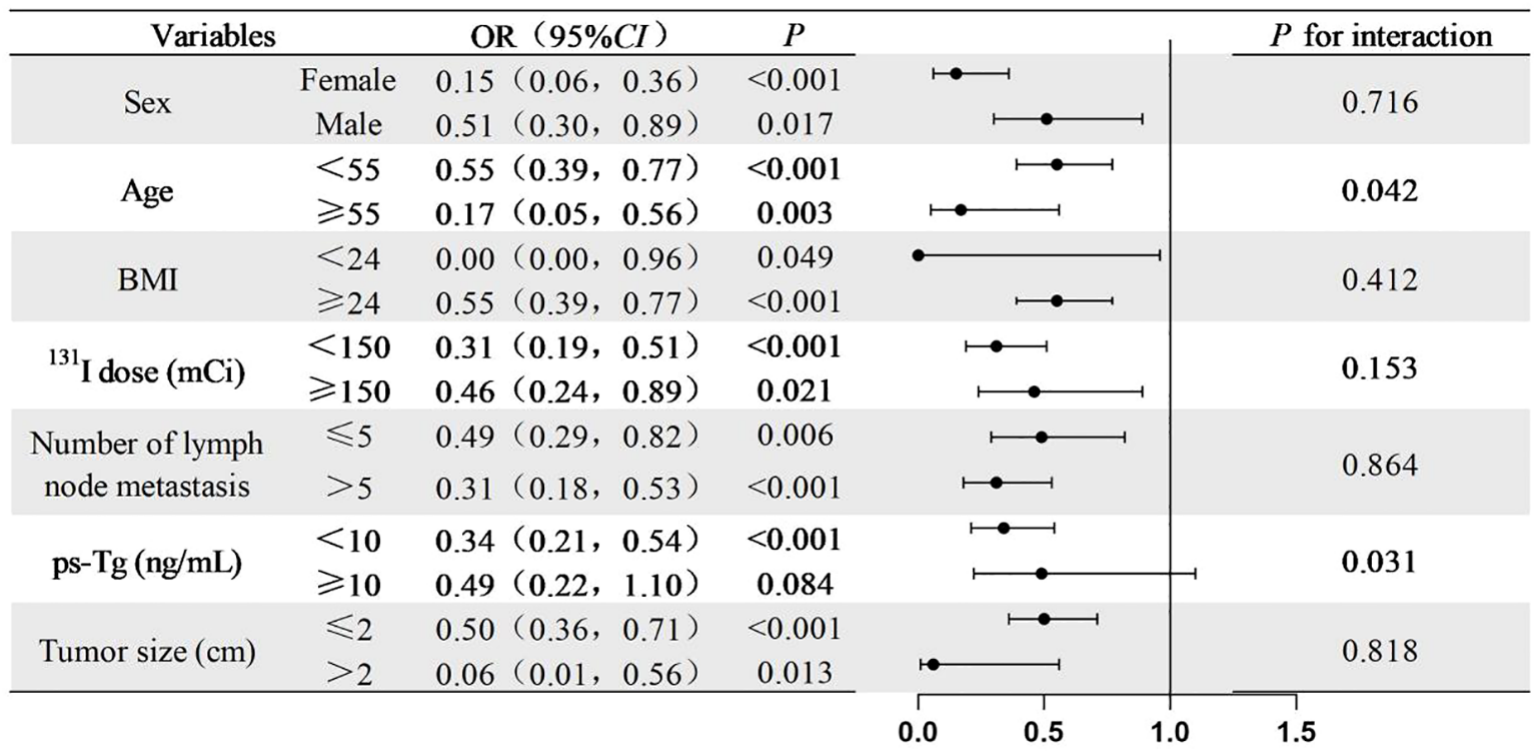
Subgroup analysis and interaction tests regarding the association between MPV and treatment response (Note: The adjustment variables are the same as in Model 2 of the multivariate logistic regression.).

Interaction tests and subgroup analyses were conducted to evaluate the stability of the association between MPV and treatment response. The results showed a significant interaction between age and MPV (*P* for interaction = 0.042), indicating that age may modify the association between MPV and treatment response. Similarly, ps-Tg showed a significant interaction with MPV (*P* for interaction = 0.031), indicating that ps-Tg may also influence the association between MPV and treatment response. No significant interactions were observed between MPV and sex, BMI, ^131^I dose, or the number of lymph node metastases. Subgroup analysis showed that the negative association between MPV and non-ER was generally consistent across subgroups. Among patients aged <55 years, each 1-fL increase in MPV was associated with a 45% reduction in the odds of non-ER (OR = 0.55, 95% CI 0.39–0.77, *P* < 0.001). Among patients aged ≥55 years, each 1-fL increase in MPV was associated with an 83% lower odds of non-ER (OR = 0.17, 95% CI 0.05–0.56, *P* = 0.003), suggesting a stronger protective association in this subgroup. In patients with ps-Tg < 10 ng/mL, each 1-fL increase in MPV was associated with a 66% reduction in the odds of non-ER (OR = 0.34, 95% CI 0.21–0.54, *P* < 0.001). In patients with ps-Tg ≥ 10 ng/mL, the association between MPV and treatment response was not statistically significant (OR = 0.49, 95% CI 0.22–1.10, *P* = 0.084). These findings suggest that the protective association between MPV and non-ER may be more pronounced in patients aged ≥55 years and in those with ps-Tg <10 ng/mL. The results are shown in **Figure 3**.

### Predictive value of MPV for ^131^I treatment response

**Figure 4:**
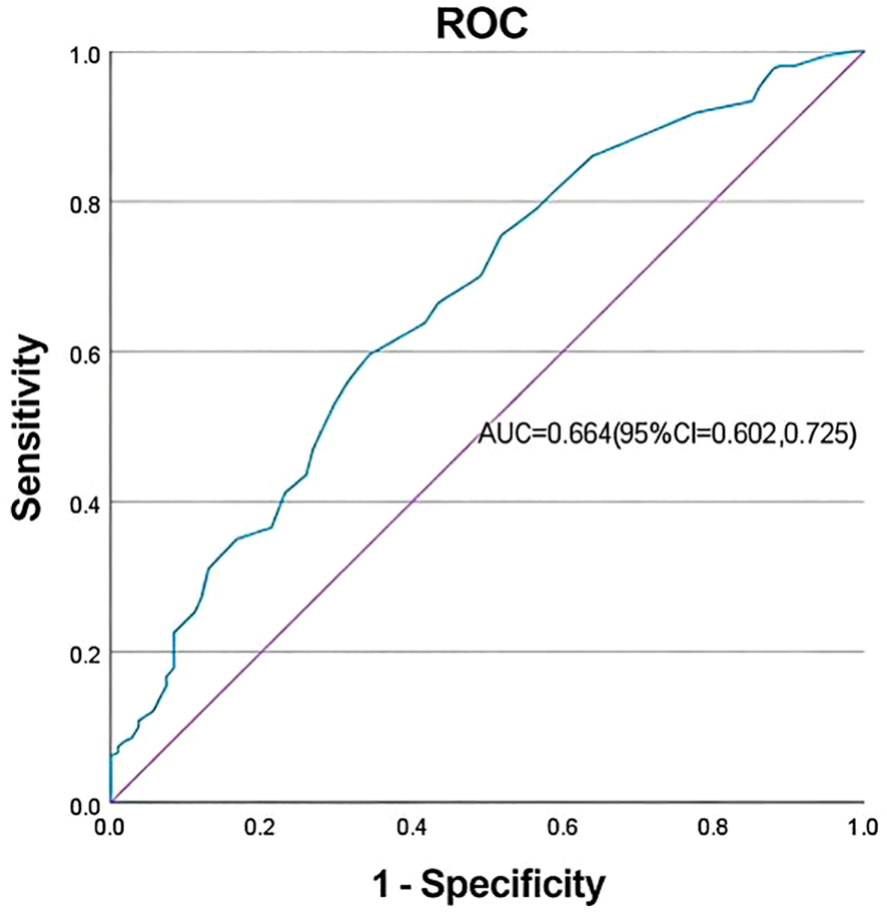
Receiver Operating Characteristic (ROC) Curves for MPV.

The ROC analysis results indicated that MPV showed moderate predictive value for the response to initial ^131^I therapy in patients with DTC, with an AUC of 0.664 (95% CI: 0.602–0.725). Using the maximum Youden index (Youden index = 0.252), the optimal cutoff value was determined to be 9.95 fL, with a sensitivity of 0.595 and a specificity of 0.657. The results are shown in **Figure 4**; **Table 4**.

**Table 4:** ROC analysis showed the value of MPV in predicting the response to ^131^I therapy.

Variables	P	AUC	95%CI	Cut-off Value	Youden index	Sensitivity	Specificity
MPV	P < 0.001	0.664	0.602, 0.725	9.95	0.252	0.595	0.657

**Figure 5:**
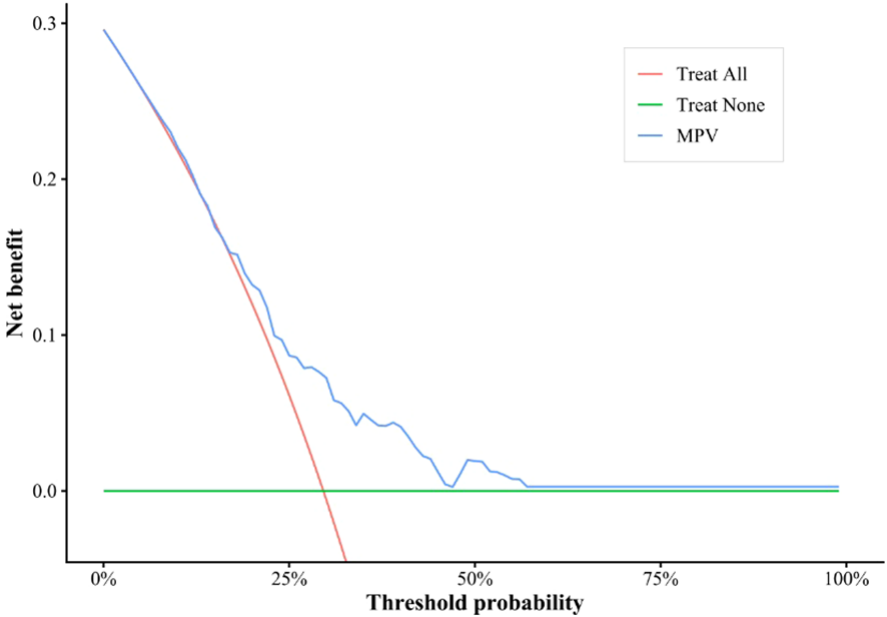
Decision Curve Analysis of MPV.

DCA was used to evaluate the clinical utility of MPV for predicting treatment response. A total of 365 participants were included in this study, among whom 108 were classified as non-ER, corresponding to a non-ER rate of 29.6%. The plot included two reference strategy lines. “Treat None” indicates that no intervention is performed for any patient, with a constant net benefit of 0. “Treat All” indicates that intervention is performed for all patients, and its net benefit generally decreases as the threshold probability increases. The results showed that MPV provided a higher net benefit than the “Treat-all” and “Treat-none” strategies across most threshold probability ranges, suggesting that MPV may have potential clinical value in assisting the assessment of treatment response. The results are shown in **Figure 5**.

## Discussion

This study focused on the association and predictive value of platelet parameters measured before initial ^131^I treatment in patients with DTC. After adjusting for potential confounding factors, MPV showed a negative association with non-ER (OR = 0.40), suggesting that higher MPV levels may be associated with lower odds of non-ER. Interaction tests suggested potential effect modification by age (*P* for interaction = 0.042) and ps-Tg level (P for interaction = 0.031). However, given the multiple comparisons conducted, particularly in the subgroup and interaction analyses, these findings should be interpreted with caution. Across most subgroups, MPV tended to be negatively associated with non-ER, although the strength of the association varied across age and ps-Tg subgroups. Regarding predictive performance, MPV showed modest discriminatory ability for predicting the response to initial ^131^I therapy in patients with DTC (AUC = 0.664). DCA suggested the potential clinical utility of MPV. Although MPV alone may be insufficient as an independent predictive tool, it may have potential value as a supplementary biomarker.

MPV, a maker of platelet size, has been reported to reflect platelet activation and systemic inflammatory status, and has been associated with the pathophysiology, severity assessment, and prognosis of various diseases (19, 20). Inflammation may partly explain the negative association between MPV and non-ER observed in this study. In chronic low-grade systemic inflammation, inflammatory cytokines, including IL-6 and IL-8, may be elevated. These cytokines may promote platelet activation and the release of larger platelets, resulting in increased MPV. In contrast, in more severe systemic inflammation, MPV may decrease due to the selective consumption of larger, highly reactive platelets and an increased proportion of smaller circulating platelets (20, 21). Therefore, lower MPV levels before ^131^I therapy may reflect a relatively more pronounced systemic inflammatory status. Previous studies have suggested that ^131^I therapy may induce inflammatory responses and immune-related changes, which could further aggravate pre-existing inflammation (22). Inflammation has also been implicated in cancer progression and tumor immune evasion (23). For example, inflammatory mediators may reshape the composition and function of immune cells in the tumor microenvironment and promote an immunosuppressive state. Under inflammatory stimulation, tumor-associated macrophages may polarize toward the M2-like phenotype and secrete immunosuppressive factors, such as IL-10 and TGF-β, thereby weakening antitumor immune responses and facilitating tumor immune evasion (24). Therefore, these mechanisms may provide a possible biological explanation for the observed association between lower MPV and non-ER.

The interaction test suggested that age may modify the association between MPV and treatment response (*P for interaction* = 0.042). Specifically, among patients aged ≥ 55 years, the negative association between MPV and non-ER was stronger (OR = 0.17). This finding may be explained by several factors. First, it may be related to age-associated immune alterations and chronic low-grade inflammation. With advancing age, the immune system gradually declines (25). An aging immune system can lead to chronic inflammation in the body. Inflammation and the aging process reinforce each other, creating a vicious cycle (26, 27). In this context, patients aged ≥ 55 years may have a higher inflammatory burden or impaired immune regulation. Furthermore, previous studies suggested that patients with DTC aged ≥ 55 years have reduced infiltration of cytotoxic CD8+ T cells in the tumor microenvironment, which may suggest a weakened antitumor immune response (28). In the setting of age-related immune dysfunction, tumor immune evasion becomes even more pronounced. Second, metabolic differences across age groups may also influence MPV levels. Previous studies have shown that MPV tends to be higher in older adults (29). These factors may partly explain why the negative association between MPV and non-ER was more pronounced in patients aged ≥ 55 years. However, the results of the above subgroup analysis should be interpreted with caution, as the sample size in the ≥ 55 years subgroup was relatively small (n = 69). In summary, age may be an important stratification variable when evaluating the association between MPV and treatment response. Nevertheless, the robustness of these findings requires further validation in future large-scale prospective studies.

The ps-Tg may also influence the association between MPV and treatment response (P for interaction = 0.031). Subgroup analysis showed that among patients with ps-Tg < 10 ng/mL, MPV was significantly and negatively associated with non-ER (OR = 0.34). In contrast, this association was not statistically significant among patients with ps-Tg ≥10 ng/mL (*P* = 0.084). When ps-Tg levels are elevated, treatment response may be more strongly influenced by the biological characteristics of the disease itself. This may attenuate or obscure the association between MPV and treatment response. The ps-Tg was closely associated with the differentiation status of tumor cells and iodine uptake, and iodine uptake is one of the key factors influencing the response to ^131^I therapy (30, 31). Previous studies have also shown that aggressive tumor characteristics are often associated with higher ps-Tg levels (32). It is worth noting that, although the interaction test reached statistical significance, the association between MPV and non-ER was not statistically significant in the ps-Tg ≥ 10 ng/mL subgroup (*P* = 0.084). This may be due to an insufficient sample size or a lack of positive events. Therefore, the robustness of this interaction requires further validation in future studies with larger sample sizes.

Several limitations of this study should be acknowledged. First, as a retrospective, single-center study, it may be subject to potential selection bias and information bias. Second, the sample size was imbalanced between the ER and non-ER groups, which may have affected the precision and stability of the estimated associations. Furthermore, the relatively small number of non-ER patients limited the feasibility of subgroup analyses for BIR, SIR, and IDR. The heterogeneity within the non-ER group may also have weakened or obscured the associations between platelet indices and specific treatment response subtypes. Therefore, future prospective multicenter studies with larger sample sizes and more balanced group distributions are needed to validate our findings.

## Conclusion

This study evaluated treatment response after initial ^131^I therapy in patients with DTC and examined the association between platelet parameters and treatment response, as well as their predictive value. The results showed that higher MPV was associated with lower odds of non-ER. As a low-cost biomarker, MPV may have adjunctive value in the early assessment of treatment response in patients with DTC following initial ^131^I therapy. These findings require further validation in larger-scale prospective studies.

## Data Availability

The raw data supporting the conclusions of this article will be made available by the authors, without undue reservation.
