# Supplementary material for: The predictive value of platelet parameters for the response to initial 131I therapy in patients with differentiated thyroid cancer
Source: Front Endocrinol (Lausanne). 2026 Jun 19;17:1850386. doi: 10.3389/fendo.2026.1850386 (PMC13327859; doi:10.3389/fendo.2026.1850386)
Supplement: Supplementary file 1 [file Table1.docx]

Supplementary Table 1. Distribution of ER and non-ER subcategories in the study cohort

| Treatment response |  | N | Percentage of total cohort (%) | Percentage of non-ER group (%) |
| --- | --- | --- | --- | --- |
| ER |  | 257 | 70.41 | — |
| non-ER |  | 108 | 29.59 | 100.00 |
|  | BIR | 24 | 6.58 | 22.22 |
|  | SIR | 31 | 8.49 | 28.70 |
|  | IDR | 53 | 14.52 | 49.07 |

Supplementary Table 2. Multicollinearity analysis

| Variable | Tolerance | VIF |
| --- | --- | --- |
| Age | 0.529 | 1.889 |
| Gender | 0.789 | 1.268 |
| BMI | 0.849 | 1.178 |
| ^131^I dose | 0.671 | 1.490 |
| Time interval | 0.947 | 1.056 |
| Tumor size | 0.559 | 1.790 |
| Cancer position | 0.790 | 1.266 |
| Extrathyroidal extension | 0.256 | 3.899 |
| Multifocality cancer | 0.809 | 1.236 |
| Lymph node metastasis | 0.720 | 1.390 |
| Number of lymph node metastasis | 0.616 | 1.624 |
| T stage | 0.199 | 5.015 |
| AJCC stage | 0.600 | 1.665 |
| ATA risk of recurrence | 0.665 | 1.503 |
| Diabetes | 0.861 | 1.162 |
| Hypertension | 0.693 | 1.442 |
| CVD | 0.828 | 1.208 |
| TPOAb | 0.956 | 1.046 |
| TgAb | 0.898 | 1.113 |
| ps-Tg | 0.764 | 1.308 |
| PLT | 0.041 | 24.247 |
| PDW | 0.837 | 1.195 |
| MPV | 0.249 | 4.016 |
| PCT | 0.049 | 20.357 |
